# Identification and characterization of lncRNA AP000253 in occult hepatitis B virus infection

**DOI:** 10.1186/s12985-021-01596-y

**Published:** 2021-06-10

**Authors:** Qingqin Hao, Zheng Wang, Qinghui Wang, Bo Chen, Huizhong Qian, Xiao Liu, Hong Cao, Wei Xia, Jian Jiang, Zhonghua Lu

**Affiliations:** 1 Department of Clinical Laboratory, Wuxi Red Cross Blood Center, 109 Xinmin Road, Wuxi, 214000 China; 2 Department of Liver Disease, Wuxi No.5 People’s Hospital Affiliated to Jiangnan University, 1215 Guangrui Road, Wuxi, 214000 China

**Keywords:** Hepatitis B virus, Occult infection, lncRNAs, AP000253, Biomarker

## Abstract

**Background:**

Recent studies suggest that lncRNAs may play significant roles in the development of hepatitis B virus (HBV) infection. However, as a special stage of HBV infection, the lncRNA expression in occult HBV infection (OBI) remains unclear.

**Methods:**

The plasma level of 15 HBV infection-related lncRNAs was initially detected using qRT-PCR in 10 OBI and 10 healthy controls (HCs) in discovery phase. Significantly dysregulated lncRNAs were subsequently validated in another 64 OBI, 20 HCs, 31 chronic hepatitis B (CHB) and 20 asymptomatic HBsAg carriers (ASC). Moreover, the AP000253 expression in liver tissues and its potential biological functions in HBV infection were further investigate with public transcriptomic data and HBV-expressing cell lines.

**Results:**

Among candidate lncRNAs, the plasma level of AP000253 decreased significantly in OBI, ASC and CHB patients compared to HCs, while no difference was found among OBI, ASC and CHB patients. In liver tissues, similar AP000253 expression was also observed from the GSE83148 dataset, while that in HBV-expressing hepatoma cells was opposite. ROC curve analysis indicated that plasma AP000253 yielded an AUC of 0.73 with 60% sensitivity and 75% specificity when differentiating OBI from HCs, but it could not specifically separate the stage of chronic HBV infection. Furthermore, functional experiments suggested that AP000253 could promote HBV transcription and replication in hepatoma cell lines.

**Conclusions:**

AP000253 might be involved in HBV replication, and be served as a potential biomarker for HBV infection. In the setting of blood donations, plasma AP000253 would be more useful to moderately distinguish OBI in HBsAg-negative donors. However, the AP000253 expression in liver tissues and associated molecular mechanism of HBV infection deserve further study in future.

**Supplementary Information:**

The online version contains supplementary material available at 10.1186/s12985-021-01596-y.

## Introduction

As a phase of chronic hepatitis B virus (HBV) infection, occult HBV infection (OBI) is characterized as an absence of serum HBV surface antigen (HBsAg) and the presence of replication-competent HBV DNA in the liver [1, 2]. The prevalence of OBI varies tremendously across the world and patient populations, with up to 0.5% in HBsAg-negative blood donors [1]. Although the precise pathogenesis is still unclear, OBI is primarily considered as the combined result of host immune control and different genomic expressions of the virus, leading to a virological quiescent state [1, 3, 4]. Hence, the blood HBV DNA is usually very low (< 200 IU/ml). Unfortunately, due to limited sensitivity of available commercial assays, viral DNA is always not or intermittently detected at this low load, also known as sampling effect of Poisson distribution [5]. Despite low viral load, OBI can still result in HBV transmission via blood transfusion, maternal and infant, organ transplant and other ways, and HBV reactivation after receiving chemotherapy or other immunosuppressive therapies, and even evolves toward serious liver diseases, such as cirrhosis, liver failure and hepatocellular carcinoma [1, 6, 7]. Therefore, owing to the potential of missed diagnosis and HBV transmission and reactivation, OBI has recently gained more and more widespread attention worldwide [8, 9].

Long noncoding RNAs (lncRNAs), used to be considered as “transcriptional noise”, are a class of transcripts longer than 200 nucleotides without protein-coding functions [10]. It is now clearly elucidated that lncRNAs may function as promising regulators of epigenetic, transcriptional and posttranscriptional gene expression, and play significant roles in various biological and pathological processes by interacting with RNA, DNA or protein and closely associated with cancers, immune, inflammatory and infectious diseases especially viral infection [11, 12]. During infection, in response to viral transcription or replication, infected cells may express viral, cellular or chimeric lncRNAs [12, 13]. Then, these lncRNAs can function diverse effects on the virus-host interaction, and consequently determine the clinical outcome [12, 14]. Up to now, several lncRNA profiles of different stages of chronic HBV infection have been reported [15–19], and functional and mechanism studies further reveal that abnormally expressed lncRNAs might play important roles in the development of HBV infection, such as HULC [19, 20], PCNAP1 [21], HOTTIP [22], lncRNA#32 [23], HOTAIR [24] and HEIH [19]. However, to our knowledge, unlike miRNAs [25–27], the lncRNA expression in OBI has been not yet investigated.

In the present study, based on our previous microarray data, public GSE83148 dataset and published studies, 15 HBV infection-related lncRNAs were selected as candidates to analyze their plasma expression in OBI and the potential as a biomarker for OBI was evaluated. Furthermore, we further investigated the AP000253 expression in tissues and its biological functions in HBV replication. Overall, these findings might provide a new insight into the screening of OBI and a better understanding of the molecular pathogenesis of HBV infection.

## Materials and methods

### Participants and specimens collection

This study was conducted in accordance with the Declaration of Helsinki and was approved by the Ethics Committee of Wuxi Red Cross Blood Center. Written informed consent was obtained from all participants.

Totally, 74 OBI, 20 asymptomatic HBsAg carriers (ASC) and 30 healthy controls (HCs) were enrolled from Wuxi red cross blood center between November 2017 and September 2020. Meanwhile, 31 patients with chronic hepatitis B (CHB) were consecutively recruited from Wuxi No.5 People's Hospital from May to September 2020. These participants were then randomly divided into the discovery cohorts (10 OBI and 10 HCs) and the validation cohorts (64 OBI, 20 ASC, 31 CHB and 20 HCs). The diagnosis of CHB, ASC and OBI was based on clinical and laboratory results according to the “Guidelines for prevention and treatment of chronic hepatitis B, China (2019)” [28] and “Update of the statements on biology and clinical impact of occult hepatitis b virus infection” [1]. Patients co-infection with hepatitis A/C/D/E virus, human immunodeficiency virus, syphilis or other serious liver diseases were excluded from this study. Blood samples were collected in ethylene-di-amine-tetra-acetic acid containing tubes (BD). After centrifuged at 3000 rpm for 20 min, plasma samples were collected and stored at − 80 °C for the following research.

### RNA extraction and qRT-PCR assay for lncRNA expression

To search for potential lncRNA biomarkers for OBI, the plasma level of 15 HBV infection-related lncRNAs were preliminarily analyzed by real-time quantitative RT-polymerase chain reaction (qRT-PCR) assay. Of these lncRNAs, 7 lncRNAs were from our previous lncRNA microarray data in liver tissues with HBV infection (AL355102.1, AL160408.1, AC022898.1, G060477, LINC00994, AC092168.1, DHRS) [18], 3 from the public GSE83148 dataset (AP000253, HNF4A-AS1 and HCP5) and 5 from published studies repeatedly reported to be associated with HBV infection (HULC, MALAT1, HOTAIR, HEIH and DREH).

lncRNA quantification was performed as previously described [18]. Briefly, total RNA was extracted with the Trizol reagent (Invitrogen, CA, USA) and the quality was assessed by NanoDrop ND1000 spectrophotometer (Agilent Technologies, CA, USA). Reverse transcription reaction and qPCR were respectively performed using SuperScriptTM III Reverse Transcriptase Kit (Invitrogen, CA, USA) and 2X SG Fast qPCR Master Mix on the LightCycler480 II Real-time PCR System (Roche, Rotkreuz, Switzerland) according to the instruction supplied by the manufacturer. The expression level of lncRNA was normalized to that of β-actin, and data were analyzed according to the comparative cycle threshold (CT) method (2^−ΔΔCT^). The sequences of the primers for lncRNAs were listed in Additional file 1: Table S1.

### Publicly available transcriptomic data analysis

The expression level of AP000253 in liver tissues from CHB patients was reviewed in the Gene Expression Omnibus database. Consequently, the hepatic transcription profile of 59 CHB patients (ALT > 40 and HBV DNA: positive) and 6 healthy controls from GSE83148 dataset (Affymetrix Human Genome U133 Plus 2.0 Array) was downloaded for the following analysis. Data was preprocessed with limma package in R language. FDR (false discovery rate) < 0.05 and |log_2_ FC|> 1(FC, Fold change) was considered statistically significant.

### Cell culture, plasmids and transfection

Human hepatoma cell lines Huh7, HepG2 and HepG2.2.15 were purchased from the Cell Resource Center (IBMS, China). All cells were cultured in DMEM (Gibco, USA) with 10% FBS (Hyclone, USA), 100 U/ml penicillin and 100 µg/ml streptomycin and maintained in a humidified atmosphere at 37 °C with 5% CO2. The pHBV1.3 plasmid containing 1.3 copies of the HBV genome was purchased from Fenghui Biological Co., Ltd (Changsha, China). The full-length AP000253 (pcDNA3.1/AP000253), short interferon RNA (siRNA) targeting AP000253 (si-AP000253) and non-targeting siRNA (si-NC) were synthesized, constructed and purchased from Genecreate Biological Co., Ltd (Wuhan, China). Transfection was conducted with Lipofectamine 2000 reagent (Invitrogen, CA, USA) according to the manufacturer’s protocols. Forty-eight hours after transfection, the cells were harvested to detect the lncRNA expression.

### HBV DNA, HBsAg and HBeAg detection

Cell culture medium were collected at 72 h after transfection and centrifuged at 2500 rpm for 5 min before the following detection. The HBV DNA in the supernatants were extracted and quantified using a diagnostic kit for quantification of HBV DNA (Kehua, Shanghai, China) on Bio-rad Real-Time PCR detection system according to the manufacturer’s instructions. While HBsAg and HBeAg were detected with a commercial enzyme-linked immunosorbent assay (ELISA) kit (Wantai, Beijing, China).

### Data analysis

All statistical analyses were performed with GraphPad Prism 5 (GraphPad Software, Inc., San Diego, USA). Continuous variables were expressed as means ± SD, while categorical data was presented as counts and percentages. Student’s *t* test or χ^2^ test was used to compare the differences between two groups for continuous or categorical variables accordingly. The relationships between AP00253 and clinical parameters were assessed by Pearson’s correlation coefficient analysis. Additionally, receiver-operating characteristic (ROC) curve was performed to evaluate the diagnostic performance of AP00253 for OBI. A value of *P* < 0.05 was considered statistically significant.

## Results

### Demographics of study population

The detailed clinical information for all subjects was presented in Table 1. Similar demographics were found among these groups. OBI patients were mainly middle-aged men. Of 34 OBI with complete serological results, 30 OBI were “seropositive OBI” including 28 Anti-HBc positive and 7 Anti-HBs positive. In addition, compared with ASC group, the viral load was lower in OBI.

**Table 1:** Baseline characteristics of the subjects in study

	Discovery phase			Validation phase				
	HCs (n = 10)	OBI (n = 10)	*P*	HCs (n = 20)	OBI (n = 64)^a^	ASC (n = 20)	CHB (n = 31)^b^	*P*
Demographic characteristics								
Female (n, %)	1 (10.00)	2 (20.00)	0.53	6 (30.00)	22 (34.38)	7 (35.00)	10 (41.94)	0.98
Age (years)	39.30 ± 11.68	42.80 ± 7.96	0.45	38.00 ± 7.94	42.86 ± 8.81	36.00 ± 7.48	43.11 ± 14.16	0.09
Serological characteristics								
ALT (U/L)	19.56 ± 7.63	22.70.67 ± 11.14	0.48	16.74 ± 6.41	18.99 ± 9.79	37.80 ± 8.23	219.94 ± 263.70	0.13
HBsAg (S/CO, positive)	0.31 ± 0.30 0	0.12 ± 0.16 0	0.12	0.21 ± 0.25 0	0.16 ± 0.21 0	22.89 ± 9.91 20	272.28 ± 139.62 31	**< *0.01***^c^
Anti-HBs (S/CO, positive)	7.00 ± 8.32 5	0.87 ± 0.72 3	***0.04***	8.24 ± 8.18 12	0.77 ± 1.21 7	0.21 ± 0.24 0	0.23 ± 0.72 0	**< *0.01***^c^
HBeAg (S/CO, positive)	0.08 ± 0.03 0	0.07 ± 0.02 0	0.89	0.07 ± 0.01 0	0.05 ± 0.02 0	0.06 ± 0.01 0	38.91 ± 82.97 7	0.46^c^
Anti-HBe (S/CO, positive)	1.60 ± 0.31 0	1.28 ± 0.47 2	0.11	1.88 ± 0.12 0	0.97 ± 0.40 8	0.24 ± 0.34 19	8.53 ± 30.90 24	**< *0.01***^c^
Anti-HBc (S/CO, positive)	1.48 ± 0.47 1	0.71 ± 0.73 7	***0.02***	1.96 ± 0.40 0	0.45 ± 0.53 28	0.08 ± 0.07 20	11.03 ± 1.84 31	**< *0.01***^c^
HBV-DNA (Ct)	NA	34.89 ± 1.25	NA	NA	35.86 ± 1.87	33.67 ± 3.01	23 ~ 2.88E + 8	**< *0.01***^c^

*OBI* occult HBV infection, *CHB* chronic hepatitis B, *ASC* asymptomatic HBsAg carriers, *HC* healthy controls, *HBsAg* hepatitis B surface antigen, *Anti-HBs* antibody for HBsAg, *HBeAg* hepatitis B envelope antigen, *Anti-HBe* antibody for HBeAg, *Anti-HBc* antibody for hepatitis B core antigen, *ALT* alanine aminotransferase, *Ct* cycle threshold, *NA* not available

^a^Due to limited volume, the results of Anti-HBs, HBeAg, Anti-HBe and Anti-HBc were only available for 34 OBI samples

^b^HBsAg, Anti-HBs, HBeAg, Anti-HBe and Anti-HBc were measured with Anytest-2000 TRF detection system, and HBV DNA was detected with HBV DNA PCR-fluorescence quantitative diagnostic kit(KHB)

^c^*P* value indicated the statistic differences between HCs, OBI and ASC groups

### Expression of 15 HBV infection-related lncRNAs in discovery phase

**Fig. 1:**
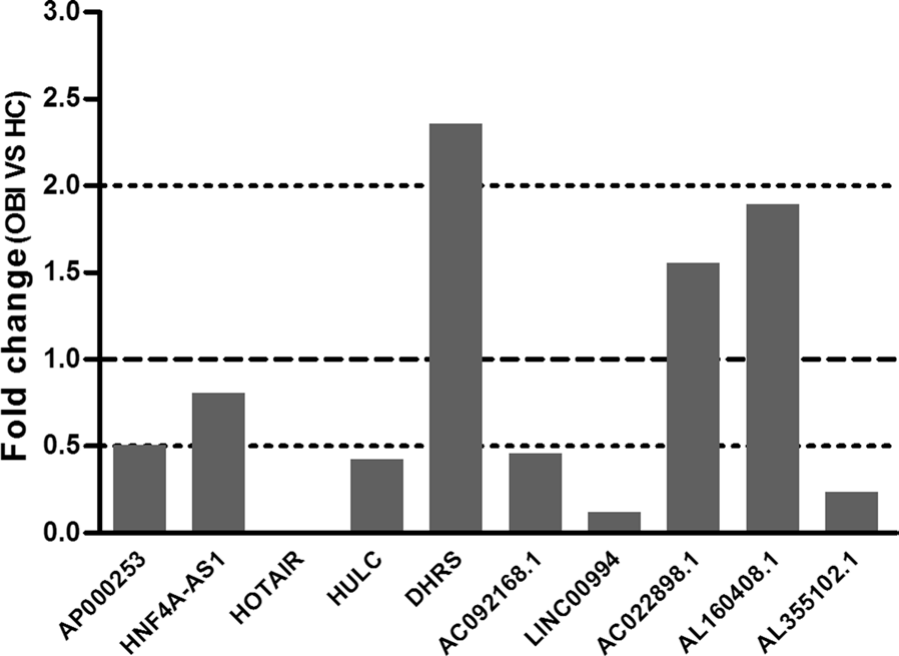
The expression level of plasma lncRNAs between OBI and HCs in the discovery cohorts. The fold change (FC) of OBI versus HC represented the ratio of the mean of target lncRNA expression between OBI and HCs groups. The expression level of lncRNAs was relative to β-actin. FC value ≤ 0.5: downregulated; FC value ≥ 2.0: upregulated; 0.5 < FC value < 2.0: no changes. *OBI* occult HBV infection, *HCs* healthy controls

The plasma level of 15 HBV infection-related lncRNAs in 10 OBI and 10 HCs were measured with qRT-PCR assay. As Fig. 1 displayed, HOTAIR, LINC00994, AL355102.1, HULC and AP000253 were significantly down-regulated, while DHRS was up-regulated in OBI when compared to HCs (FC > 2). No significant differences were observed in the level of HNF4A-AS1, AC092168.1, AC022898.1 and AL160408.1. Moreover, the remaining 5 lncRNAs, namely HCP5, MALAT1, HEIH, DREH and T262735, failed to be detected in plasma. Collectively, except DHRS because of low expression level, HOTAIR, LINC00994, AL355102.1, HULC and AP000253 were selected as candidate lncRNAs for further validation.

### Validation of 5 candidate lncRNAs in plasma by qRT-PCR

**Fig. 2:**
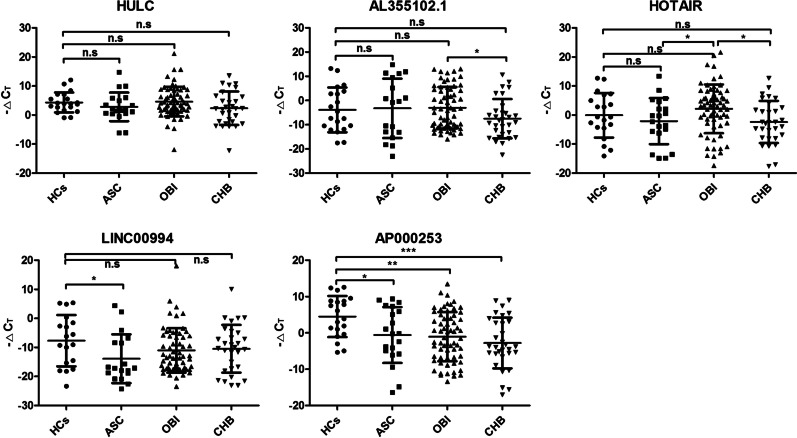
The expression level of 5 candidate lncRNAs in plasma among study subjects in the validation cohorts. The relative expression of lncRNA was reported as ΔCT, which was calculated by subtracting the CT (cycle threshold) of β-actin from the CT of target lncRNAs. *OBI* occult HBV infection, *CHB* chronic hepatitis B, *ASC* asymptomatic HBsAg carriers, *HCs* healthy controls; ***P < 0.001; **P < 0.01; *P < 0.05, *n.s.* not significant

Then, the plasma level of 5 candidate lncRNAs were further validated in independent cohorts including 20 HCs, 64 OBI, 31 CHB and 20 ASC. As shown in Fig. 2. The level of AP000253 was significantly lower in OBI, ASC and CHB groups compared with HCs group, and gradually increased from CHB to OBI, ASC and HCs, despite no significant difference was found among OBI, ASC and CHB (*P* > 0.05). Additionally, the level of LINC00994 was also lower in ASC than in HCs group. When compared to OBI, the lower level of HOTAIR in ASC and CHB groups was observed. Beside these differences, the level of these 5 lncRNAs was not significantly altered between other two groups.

### Associations between plasma AP000253 and clinical characteristics

**Fig. 3:**
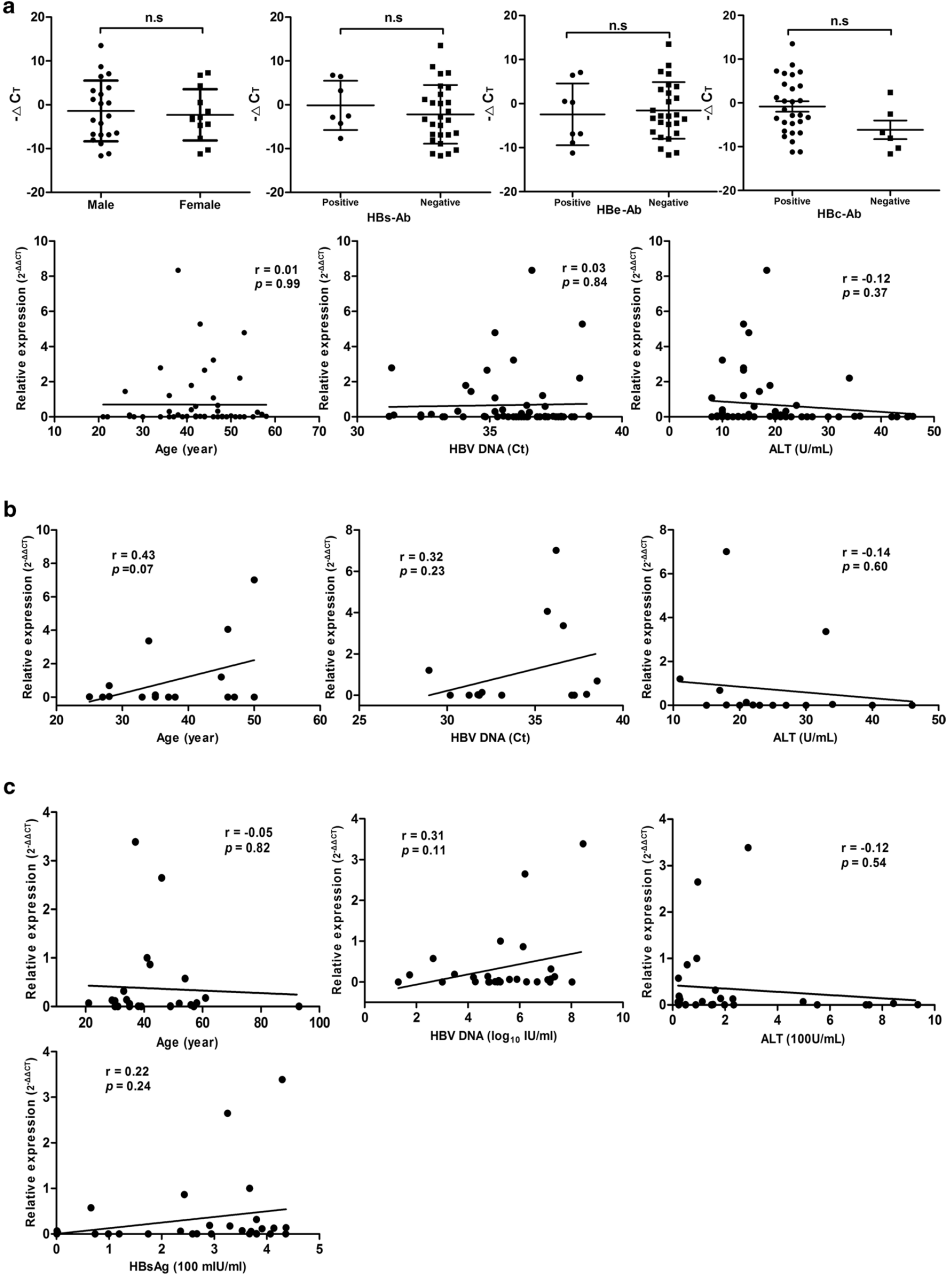
Correlations between plasma AP000253 and clinical characteristics of patients with HBV infection. Correlations between plasma AP000253 and sex, HBs-Ab, HBe-Ab, HBc-Ab, age, HBV DNA and ALT in OBI group (**A**); Correlations between plasma AP000253 and age, HBV DNA and ALT in ASC group (**B**); and correlations between plasma AP000253 and age, HBV DNA, ALT and HBsAg in CHB group (**C**). The correlation co-efficiency (r) and the two-tailed *P* values were evaluated with Pearson’s test. *OBI* occult HBV infection, *CHB* chronic hepatitis B, *ASC* asymptomatic HBsAg carriers, *ALT* alanine aminotransferase, *Ct* cycle threshold

The associations between the expression level of AP000253 and clinical characteristics were further explored and no significant association with sex, age, the serum level of ALT and serologic viral markers, including HBs-Ab, HBe-Ab, HBc-Ab and HBV-DNA, was observed in OBI group (Fig. 3A). Meanwhile, no significant association was also found between plasma AP000253 and age and the serum level of ALT, HBV-DNA or HBsAg in ASC (Fig. 3B) and CHB groups (Fig. 3C). Moreover, the detail results of correlation analysis between plasma AP000253 and serologic viral characteristics of patients with HBV infection were summarized in Additional file 2: Table S2.

### Diagnostic performance of plasma AP000253 for OBI

**Fig. 4:**
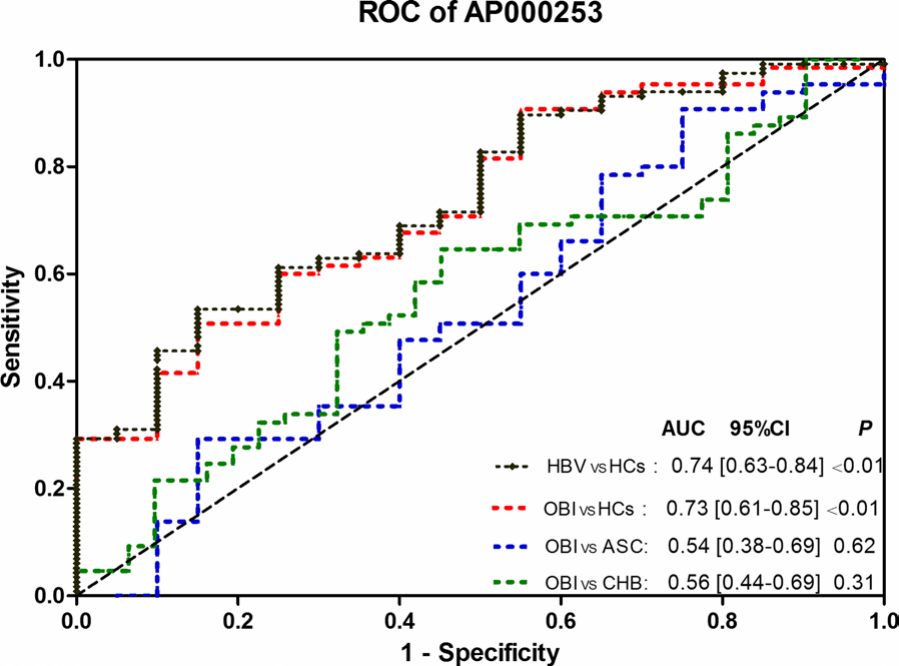
Receiver operating characteristic (ROC) curve analysis for discriminative ability among OBI, ASC, CHB and HCs by plasma AP000253 in the validation cohorts. *OBI* occult HBV infection, *CHB* chronic hepatitis B, *ASC* asymptomatic HBsAg carriers, *HBV* patients with HBV infection, *HCs* healthy controls, *AUC* the area under the ROC curve

Subsequently, the diagnostic value of plasma AP000253 for OBI was evaluated. The ROC curve analysis demonstrated that AP000253 was useful in differentiating OBI from HCs with an AUC of 0.73, at a cutoff value of − 0.84 for AP000253 expression level (ΔCT), the optimal sensitivity and specificity were 60% and 75%, respectively. Comparable differentiation power between patients with HBV infection and HCs was also achieved with the AUC of 0.74. At the cutoff value of 1.175 for AP000253 expression level (ΔCT), the optimal sensitivity and specificity were 53.45% and 85%, respectively. However, AP000253 failed to differentiate OBI from ASC or CHB patients (Fig. 4).

### lncRNA AP000253 expression in liver tissues and human hepatoma cell lines

**Fig. 5:**
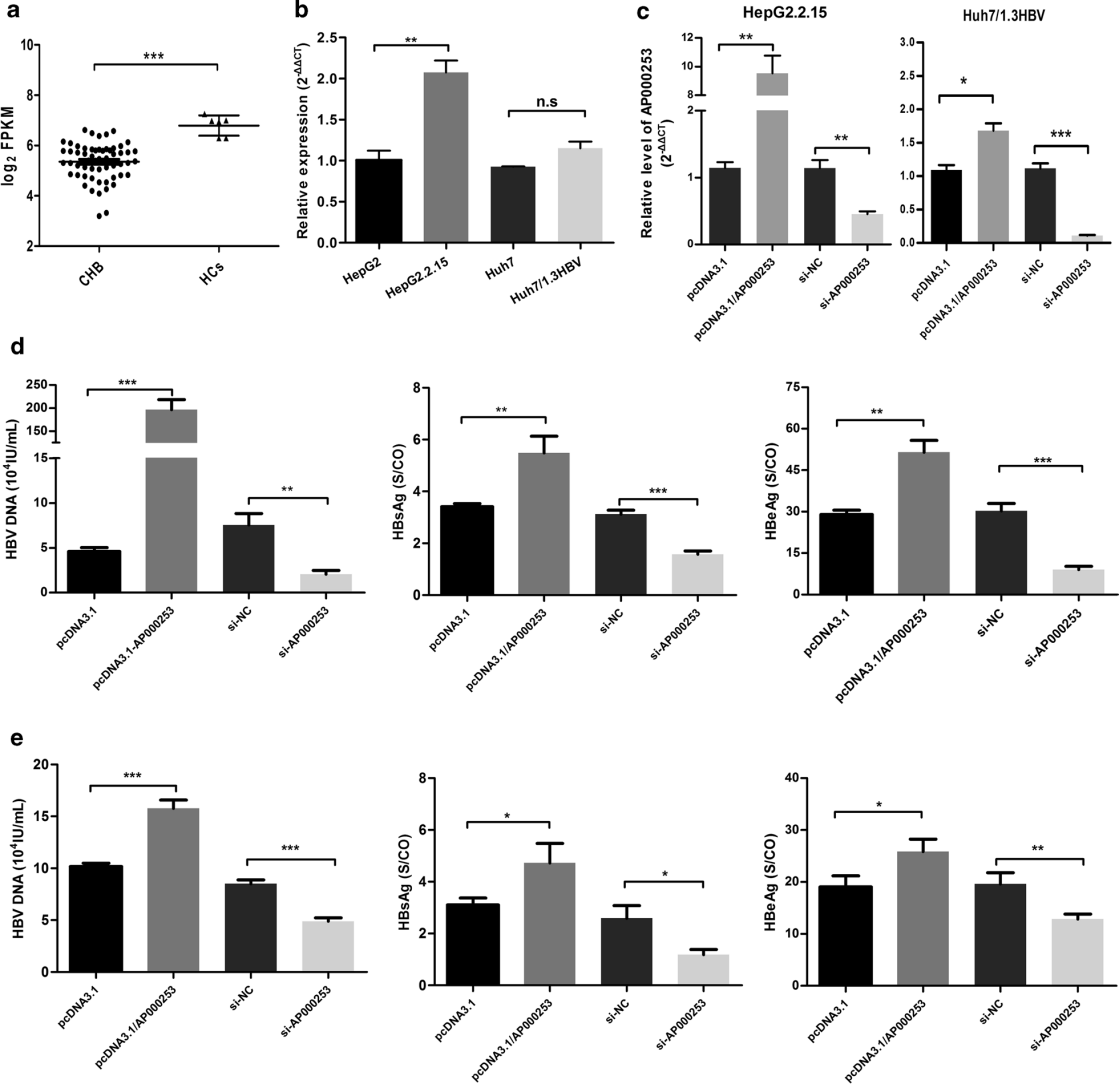
lncRNA AP000253 promoted HBV transcription and replication. (**A**) The relative level of AP00253 was determined by microarray in liver tissues of CHB (n = 59) and healthy controls (n = 6) from the GSE83148 dataset; (**B**) The level of AP00253 was detected by qRT-PCR in HepG2, HepG2.2.15, Huh7 and Huh7/pHBV1.3 cells; (**C**) Validation of overexpression and knockdown efficiency of AP000253 in both HepG2.2.15 and Huh7/pHBV1.3 cells by qRT-PCR, respectively; (**D**) The levels of HBV DNA, HBsAg and HBeAg were measured by qPCR or ELISA assays in the supernatant of HepG2.2.15 cells treated with si-NC or si-lncRNA AP000253 and pcDNA3.1 or pcDNA3.1/lncRNA AP000253 for 3 days, respectively; (**E**) The levels of HBV DNA, HBsAg and HBeAg were measured by qPCR or ELISA assays in the supernatant of Huh7/pHBV1.3 cells treated with si-NC or si-lncRNA AP000253 and pcDNA3.1 or pcDNA3.1/lncRNA AP000253 for 3 days, respectively; *CHB* chronic hepatitis B, *HCs* healthy controls; ***P < 0.001; **P < 0.01; *P < 0.05, *n.s.* not significant

According to the GSE83148 dataset, we found that the level of AP000253 was also significantly lower in liver tissues with CHB compared to normal liver tissues (log_2_FC = − 1.44, FDR = 0.0001, Fig. 5A and Additional file 3: Table S3), consistent with that in plasma. However, unexpectedly, the level of AP00253 was higher in HBV-expressing hepatoma cell lines, despite the difference did not reach statistical significance in Huh7/pHBV1.3 cell line (Fig. 5B).

### lncRNA AP000253 promoted HBV replication in human hepatoma cell lines

Finally, to gain further insight into the biological roles of AP000253 in HBV infection, AP000253 over-expression plasmid (pcDNA3.1/AP000253) and knockdown plasmid (si-AP000253) were generated and transfected into Huh7/pHBV1.3 and HepG2.2.15 cells. The over-expression and knockdown efficiency of AP000253 was further confirmed by qRT-PCR (Fig. 5C). As the results demonstrated, ELISA and qPCR assays indicated that HBV DNA, HBsAg and HBeAg increased in the supernatant of AP000253-overexpressing cells and reduced in AP000253-knockdown cells at 72 h after transfection (Fig. 5D, E). These results suggested that AP000253 might facilitate the HBV transcription and replication.

## Discussion

The existence of OBI makes the management of HBV infection more difficult, and is a serious threat to the transfusion safety [8, 9]. To improve the screening and a better understanding of the pathogenesis of OBI is therefore desirable [1, 4, 6, 29]. Recent studies have identified many lncRNAs associated with HBV infection, and an understanding of their functional roles in the pathogenesis of HBV infectious diseases is just emerging [20, 21]. However, the lncRNA expression in OBI is still unclear.

In the present study, among 15 HBV infection-related lncRNAs, only plasma AP000253 was consistently differently expressed between OBI and HCs. Despite there was a rising trend from CHB to OBI, ASC and HCs, no significant difference in plasma level of AP000253 was found among OBI, ASC and CHB groups. Subsequently, We respectively evaluated the correlation between plasma AP000253 and clinical characteristics in OBI, ASC and CHB groups, and no association with demographic characteristics or serologic viral biomarkers such as antigen, antibody and nucleic acid, was found, which indicated that plasma AP000253 might serve as an independent biomarker of infection regardless of the progression status of HBV infection. Unfortunately, due to the scarcity and limited volume of OBI samples, the associations between AP000253 and overall clinical parameters could not be systematically and comprehensively assessed.

Currently, the gold standard for the diagnosis of OBI was based on the detection of replication-competent viral DNA (i.e. cccDNA and rcDNA), not the presence of integrated viral DNA fragment in the liver [31–33]. Regrettably, a standardized assay with internal and external validity is still nonexistent yet. In clinical practice, due to the always unavailable liver tissues, the diagnosis of OBI is commonly based on the detection of HBV DNA in the blood instead. While, unfortunately, because of limited sensitivity of the existing commercial assays, OBI is often missed [5]. As reported, to prevent HBV transmission by transfusion, the sensitivity of nucleic acid assays would need to be lowered from the current 3.4 IU/ml to a new lower limit of detection of 0.15 IU/ml [6]. Hence, to identify novel biomarkers or approaches to improve the OBI screening is urgent. Recently, increasing evidence has demonstrated that lncRNAs had great potential as a diagnostic biomarker for many diseases [34, 35]. Then, the potential of AP000253 as a novel blood-based biomarker in OBI screening was evaluated. Consequently, plasma AP000253 could moderately differentiate OBI from HCs, but fail to discriminate OBI from ASC as well as CHB. When differentiating patients with HBV infection from HCs, similar performance was also observed. These results suggested that AP000253 might be useful in identifying patients with HBV infection, but fail to specifically separate the stage of chronic HBV infection. In the setting of blood donations, in order to reduce the residual risk of transfusion-transmitted HBV infection, the screening strategy adopted by Chinese blood centers is as following: (1) HBsAg rapid screening test in pre-donation, (2) if nonreactive (HBsAg negative), and then two rounds of HBsAg screening with different ELISA assays combined with nucleic acid testing in post-donation [36, 37]. In our blood center, only when HBsAg screening is nonreactive, nucleic acid testing for HBV DNA is available. Hence, in this circumstance, plasma AP000253 would be more useful and practical in discriminating OBI from HCs in HBsAg-negative populations, despite the diagnostic performance is moderate.

Like miRNAs, the lncRNA secretion may be also a selective process, and the peripheral blood level of lncRNAs is not always a true reflection of the intracellular level [38]. Therefore, we further investigated the AP000253 expression in liver tissues with CHB instead, because of the liver tissues with OBI were unavailable. Similarly, in the public GSE81348 dataset, the level of AP000253 was also significantly decreased. However, in HepG2.2.15 and HBV-transfected Huh7 cell lines, the expression level was opposite. One possible explanation for this discrepancy might be the interference of interstitial cells in liver tissue. Regrettably, owing to the limited resources of the liver tissues from CHB patients and healthy people, we failed to further experimentally confirm the AP000253 expression in liver tissues, which deserve further confirmation, especially in liver tissues with OBI.

Finally, accumulating evidence has indicated that lncRNAs can participate in diverse physiological and pathological processes and affect disparate cellular functions [11]. To the best of our knowledge, this is the first report to elaborate AP000253. AP000253 (LOC102724449), a neighbor lncRNA gene of human SOD1 gene, is located at chromosome 21q22.11 with a length of 4282 bp, containing three exons. According to the RNA-Seq expression data from GTEx database, AP000253 shows tissue-specific distributions, and is highly expressed in the liver and testis. Moreover, it has no coding probabilities predicted by the Coding Potential Calculator. The subcellular localization of lncRNAs always determines their possible mechanisms. From the lncLocator database, AP000253 transcript (XR_430363.3) is predominantly located in cytoplasm, suggesting that it may exert biological functions at post-transcriptional level. Due to the cell or animal models of OBI are not available, we then try to preliminarily explore the possible biological functions of AP000253 using HepG2.2.15 and HBV-transfected Huh7 cell lines. ELISA and qPCR assays indicated that AP000253 could significantly enhance the levels of HBsAg, HBeAg and HBV DNA, suggesting that AP000253 might be able to modulate HBV transcription and replication in hepatoma cells. However, the molecular mechanisms of AP000253 in modulating HBV replication need to be further clarified, which is what we are now working on.

## Conclusion

In conclusion, we report that AP000253 might promoted HBV transcription and replication, and decreased level of AP000253 was observed in plasma of OBI, ACS and CHB patients. In the setting of blood donations, AP000253 might be more useful as a biomarker of HBV infection to moderately distinguish OBI from HCs in HBsAg-negative donors. However, the AP000253 expression in liver tissues and AP000253-associated molecular pathological mechanism in HBV infection should be further explored in future.

## Supplementary Information

**Additional file 1**. Table S1. Primers used for quantitative PCR of lncRNAs.

**Additional file 2**. Table S2. Correlations between plasma AP000253 and clinical parameters in the validation cohorts.

**Additional file 3**. Table S3. The characteristic of differentially expressed AP00253 in chronic hepatitis B from the GSE83148 dataset.

## Data Availability

The data and material generated or analyzed in this study are available upon reasonable request, and could be provided by Zhonghua Lu (Lu_z_h1@126.com) or Wei Xia (xiawe25@126.com).

## Abbreviations

lncRNAs: Long noncoding RNAs
OBI: Occult HBV infection
CHB: Chronic hepatitis B
ASC: Asymptomatic HBsAg carriers
HBV: Hepatitis B virus
HCs: Healthy controls
GEO: Gene Expression Omnibus database
qRT-PCR: Real-time quantitative RT-polymerase chain reaction
HBsAg: Hepatitis B surface antigen
Anti-HBs: Antibody for HBsAg
HBeAg: Hepatitis B envelope antigen
Anti-HBe: Antibody for HBeAg
Anti-HBc: Antibody for Hepatitis B core antigen
ALT: Alanine aminotransferase
Ct: Cycle threshold
