# Supplementary material for: Identification and characterization of lncRNA AP000253 in occult hepatitis B virus infection
Source: Virol J. 2021 Jun 10;18:125. doi: 10.1186/s12985-021-01596-y (PMC8194241; doi:10.1186/s12985-021-01596-y)
Supplement: Supplementary file 1 — Additional file 1. Table S1. Primers used for quantitative PCR of lncRNAs. [file 12985_2021_1596_MOESM1_ESM.doc]

**Supplementary Table S1. Primers used for quantitative PCR of lncRNAs.**

| **lncRNAs** | **Primer sequence(5’3’)** |
| --- | --- |
| AL355102.1 | F: CCCCAAACCTGTTCTTCACTC  R: TTTTGTGGAGATGGGCAGTT |
| AL160408.1 | F: AATACCAGCAAGACCACCCAG  R: TTCTGGTGGAGTGGTCATCAA |
| AC022898.1 | F: CATTGCTATTGAATCGGGAAGT  R: GAGCCAAAGTCCTTCTCGTTC |
| T262735 | F: TTTCCTCTTTCCCACCTGTTT  R: TCTTGTTTCTCTTCATCTCTGCC |
| LINC00994 | F: CATCATGGCAGGTGGTTTG  R: AGGACTGTTGGGAGCATTTCT |
| AC092168.1 | F: TTTCAGACCACGAGGACACTT  R: TCTGGAATGGAGTCGGGAT |
| DHRS | F: TTCCTTGCTACTTCCTCTTTCAC  R: GCTTATCGCAGACTTGGCTC |
| HULC | F: ATCTGCAAGCCAGGAAGAGTC  R: CTTGCTTGATGCTTTGGTCTGT |
| DREH | F: GCTAACGAACAAAGCCAGA  R: CCCTATTCTCATGCAAGGA |
| HEIH | F: CCTCTTGTGCCCCTTTCTT  R: ATGGCTTCTCGCATCCTAT |
| HOTAIR | F: GGTAGAAAAAGCAACCACGAAGC  R: ACATAAACCTCTGTCTGTGAGTGCC |
| MALAT1 | F: CCAGTTGAATTCACCAGTGGAC  R: AGTTTGCTCACATGCCAGTTAC |
| HCP5 | F: AGGTGCCTATCCCTGTGAAGA  R: CCAGACCCTAAGTGAGGAGTTG |
| HNF4A-AS1 | F: GATACCAGGTCAAGGATTCGG  R: TGTGCAGTCAAGATTTAGGCC |
| AP000253 | F: GTGTTGAAGCCAAGAAGATGC  R: AGTGAGCCGAGATCATGCC |
| b-actin | F: TAGTTGCGTTACACCCTTTCTTG  R: TCACCTTCACCGTTCCAGTTT |
