# Supplementary material for: Identification and characterization of lncRNA AP000253 in occult hepatitis B virus infection
Source: Virol J. 2021 Jun 10;18:125. doi: 10.1186/s12985-021-01596-y (PMC8194241; doi:10.1186/s12985-021-01596-y)
Supplement: Supplementary file 2 — Additional file 2. Table S2. Correlations between plasma AP000253 and clinical parameters in the validation cohorts. [file 12985_2021_1596_MOESM2_ESM.doc]

**Supplementary Table S2. Correlations between plasma AP000253 and clinical parameters**

| **Variables** | ***P* value** | | |
| --- | --- | --- | --- |
|  | **OBI** | **ASC** | **CHB** |
| Sex (female/male) | 0.71 | 0.73 | 0.05 |
| HBsAg(positive/negative) | NA | NA | NA |
| Anti-HBs(positive/negative) | 0.46 | NA | NA |
| HBeAg(positive/negative) | NA | NA | 0.67 |
| Anti-HBe(positive/negative) | 0.74 | 0.74 | 0.63 |
| Anti-HBc(positive/negative) | 0.06 | NA | NA |

^*^*P* value were calculated by Student’s t test.

OBI: occult HBV infection; CHB: chronic hepatitis B; ASC: asymptomatic HBsAg carriers; HBsAg: Hepatitis B surface antigen; Anti-HBs: antibody for HBsAg; HBeAg: Hepatitis B envelope antigen; Anti-HBe: antibody for HBeAg; Anti-HBc: antibody for Hepatitis B core antigen; NA: not available.
